# Application of chromosome microarray analysis in prenatal diagnosis

**DOI:** 10.1186/s12884-020-03368-y

**Published:** 2020-11-16

**Authors:** Mingjing Xia, Xinhong Yang, Jing Fu, Zhenjuan Teng, Yan Lv, Lixia Yu

**Affiliations:** Department of Obstetrics, Weihai Maternal and Child Health Hospital, the Affiliated Weihai Second Municipal Hospital of Qingdao University, Weihai, China

**Keywords:** Chromosome microarray analysis, Karyotype analysis, Prenatal diagnosis, Ultrasound abnormalities

## Abstract

**Background:**

To explore the application value of chromosomal microarray analysis (CMA) in prenatal diagnosis.

**Methods:**

The results of chromosome karyotype analysis and CMA of 477 cases undergoing amniocentesis were analyzed. The results of the no ultrasound abnormality group and the ultrasound abnormality group were compared separately. Within the ultrasound abnormality group, the results of the ultrasound structural malformation group, the ultrasound soft index abnormality group, and other ultrasound abnormality (including abnormal amniotic fluid volume and fetal growth restriction) groups were compared.

**Results:**

Abnormal chromosome and CMA results were found in a total of 71 cases (15.88%, 71/447), which can be broken down into a total of 23 karyotype abnormalities (5.15%, 23/447), consisting of 18 cases of aneuploidy (4.03%, 18/447), 2 cases of unbalanced chromosome rearrangements (0.44%, 2/447), and 3 cases of chimerism (0.67%, 3/447); 17 cases with detection of pathogenic copy number variations (pCNVs) (3.80%, 17/447); and 31 cases of detection of clinical variants of unknown significance (VOUS) (6.93%, 31/447). CMA detected 3.8% more genetic abnormalities than karyotype analysis (in addition to the abnormalities detected simultaneously by karyotype analysis). Between the no ultrasound abnormality group and the ultrasound abnormality group, there was an extremely significant difference in the detection rate of an abnormal chromosomal karyotype (*P* < 0.01) and of VOUS (*P* < 0.01), but there was no significant difference in the detection rate of pCNV (*P* > 0.05). Comparing the ultrasound structural malformation group, the ultrasound soft index abnormality group, and the other ultrasound abnormality group, there were no significant differences in the detection rate of abnormal chromosomal karyotypes (*P* > 0.05), pCNV (*P* > 0.05) or VOUS (*P* > 0.05).

**Conclusions:**

The detection rate of chromosomal karyotype abnormalities in prenatal diagnosis in cases with no ultrasound abnormalities was higher. For cases with fetal ultrasound structural abnormalities, when compared with traditional karyotype analysis, CMA can improve the detection rate of fetal genetic abnormalities. However, the no ultrasound abnormality group also had a high VOUS abnormality detection rate, so it is necessary to strictly define the CMA indications.

## Background

Birth defects include structural abnormalities and genetic abnormalities in the fetus. Chromosome G-band karyotyping analysis is widely used as a method for detecting genetic abnormalities in clinical prenatal diagnosis. However, this approach has several limitations, including the necessity of performing cell culture, a lengthy detection wait period (2 to 3 weeks), and a low resolution (only chromosomal abnormalities > 5–10 Mb can be detected using this approach).

Chromosomal microarray analysis (CMA) is an emerging molecular genetic detection technology in the field of prenatal diagnosis. This approach can accurately detect the number and structural abnormalities of chromosomal imbalances and detect chromosomal alterations such as microdeletions and microduplications. CMA has the advantages of a high resolution, a short detection cycle, and more objective results.

Since 2009, the American Obstetrics and Gynecology Association, the Canadian Obstetrics and Gynecology Association, the European Cytogenetics Association, and China have all published guidelines recommending CMA technology as the first-line prenatal diagnostic detection method after detecting prenatal fetal ultrasound structural malformations. We analyzed the results of 477 cases that underwent interventional prenatal diagnosis for different reasons, all of which underwent both chromosome karyotype analysis and CMA detection, to explore the value of CMA in prenatal diagnosis.

## Methods patient eligibility criteria

1.1 A total of 477 pregnant women at the Weihai Maternal and Child Health Hospital who needed a prenatal diagnosis were enrolled in the study from January 2016 to December 2019. All pregnant women underwent amniocentesis to obtain fetal amniotic fluid for karyotype analysis and CMA detection. The reasons why the pregnant women voluntarily underwent interventional prenatal diagnosis and chromosome karyotype analysis plus CMA detection, which were the inclusion criteria, were as follows: (1) ultrasound abnormalities, including single-system or multisystem structural abnormalities; (2) soft ultrasound indicators, including thickened posterior neck zona pellucida, missing or short nasal bones, bright ventricular spots, enhanced bowel echo, dilated ventricles, widened posterior cranial fossa pool, choroid plexus cyst, dilated renal pelvis, or a short femur; (3) a lack of nonstructural abnormalities such as intrauterine growth retardation, polyhydramnios/oligohydramnios; or (4) no ultrasound abnormality, including women with noninvasive prenatal testing (NIPT) abnormalities, a history of adverse pregnancy outcomes, and other factors for prenatal diagnosis. This study was approved by the Scientific and Medical Ethics Committee of Weihai Maternal and Child Health Hospital (batch number: 2016-LW-26), and all pregnant women signed informed consent forms.

### Study design

Amniocentesis sample collection was performed under ultrasound-guided localization. Two 10 ml tubes of amniotic fluid were collected and stored at 4 °C. One tube was used for CMA detection, and the other was used for karyotype analysis. For amniotic fluid samples with maternal cell contamination, the CMA test was performed after the amniotic fluid cells were cultured.

Karyotype analysis culture samples were prepared according to standard cytogenic procedures for G-banding. Chromosome microarray analysis samples were extracted using a CytoScan Reagent Kit (Affymetrix, USA) according to the manufacturer’s instructions. An Affymetrix CytoScan 750 K Array (Affymetrix, USA) chip was used for whole-genome scanning. ChAS 3.2 software was used for analysis. The Affymetrix CytoScan 750 K platform contains 200,000 single nucleotide polymorphism (SNP) tags and 550,000 copy number variation (CNV) tags distributed at an average density of approximately 1 tag/4 Kb. All procedures were performed strictly in accordance with the manufacturer’s instructions.

### Interpretation of chromosome microarray analysis results

Karyotype analysis and descriptions are based on the International Human Cytogenetic Naming System (2013). All segments were monitored for the degree of overlap with previously identified common CNVs and annotated by DGV. All reported CNVs were based on the National Center for Biotechnology Information human genome build 37 (hg 19). The position of the chromosome GRCH37/hg19 is described according to the genomic version of the system. This chip detects chromosome copy number changes at a specific resolution. The samples are analyzed at a resolution level of 50 Kb/25 marker deletions and 100 Kb/25 marker repetitions. Generally, fragments smaller than 100 Kb and deletions smaller than 200 Kb were not reported. No copy number changes across the genome that are known to be normal polymorphisms were reported.

This analysis was completed with the assistance of the following databases: the Database of Genomic Variants (DGV, http://dgv.tcag.ca/dgv/), the International Standards for Cytogenomic Arrays (ISCA, http://www.iscaconsortium.org/), the Online Mendelian Inheritance in Man database (OMIM, http://www.ncbi.nlm.nih.gov/omim), the Database of Chromosomal Imbalance and Phenotype in Humans using Ensembl Resources (DECIPHER, https://decipher.sanger.ac.uk/), the University of California Santa Cruz Genomic Browser (UCSC, http://genome.ucsc.edu/), and PubMed (https://www.ncbi.nlm.nih.gov/pubmed/).

Interpretation of CNVs: CNVs > 100 Kb were classified according to the guidelines defined by the clinical significance of the CNVs [1]. According to the standards and guidelines established by the American College of Medical Genetics (ACMG) for CNV interpretation, the CNVs were divided into three categories: (1) pathogenic CNV (pCNV), (2) benign CNV (bCNV), and (3) clinical CNV (variants of unknown significance, VOUS). CNVs were classified according to the American College of Medical Genetics (ACMG) guidelines.

In each case, karyotype analysis and CMA testing were performed simultaneously. Aneuploidy, abnormal structural abnormalities and chimeras detected by karyotype, and CMA analyses were classified as chromosomal abnormalities. Microdeletions and microduplications detected only by CMA were classified as copy number variations.

### Statistical methods

SPSS 22.0 software was used for statistical analysis. The count data are expressed as both a frequency and a percentage. Comparisons between groups were performed using a χ2 test, and *P* < 0.05 was considered statistically significant.

## Results

Pregnant women enrolled in the study were aged 21 to 44 years, with an average 32.85 ± 6.51 years of age, and they were 19–33 weeks pregnant, with an average of 24.52 ± 4.73 weeks.

### Overall results

The results were normal in a total of 376 cases (84.12%, 376/447).

### Chromosomal karyotyping and CMA abnormal results

Seventy-one cases were abnormal (15.88%, 71/447), including 23 cases with chromosomal abnormalities (5.15%, 23/447), of which there were 18 cases of aneuploidy (4.03%, 18/447), 2 cases of unbalanced rearrangements (0.44%, 2/447), and 3 cases of chimerism (0.67%, 3/447); 17 cases of pCNVs (3.80%, 17/447); and 31 cases of VOUS (6.93%, 31/447). See Table 1.

**Table 1:** Results of different indications for prenatal diagnosis

Prenatal diagnostic indications	n	Abnormal karyotype	Detection rate (%)	PCNVS	Detection rate (%)	VOUS	Detection rate (%)
NIPT abnormal	25	8	32	1	4	4	16
Heart malformation	63	3	4.76	2	3.17	5	7.94
Urinary system abnormalities	56	0		3	5.35	1	1.78
Multiple malformations	9	3	33.33	0		0	
Lateral ventricle dilatation	38	1	2.63	2	5.26	1	2.63
Digestive disorders	22	0		1	4.55	2	9.09
NT > 3 mm	31	1	3.23	0		1	3.23
Hydrocyst of the neck	8	1	12.5	0		0	
Foot inversion	4	1	25	0		0	
Abnormal karyotypeof couple	8	1	12.5	0		1	12.5
Abnormal Karyotype	11	1	9.09	1	9.09	1	9.09
High-riskscreening	3	0		1		1	
Fetal growth restriction	30	1		1		1	
Oligohydramnios	2	0		1		0	
advanced maternal age	3	2					
Nervous system abnormalities	15	0		2		5	
Cleft lip and palate	5			2			
Polyhydramnios	22					3	
stillbirth	9					2	
Adverse pregnancy history	3					1	
FISH does not match karyotype	2					1	
Short nasal bone	12					1	
Left ventricular strong echo	13						
Choroid plexus cyst	11						
Strong echo of bowel	13						
Eyes wide	1						
*Pleural effusion*	*7*						
Skeletal abnormalities	11						
Pulmonary cystadenoma	5						
Diaphragmatic hernia	4						
Isolated lung	1						
Total	447	23		17		31	

Each case is classified according to its most important indication. Indications are listed in order of importance: abnormal ultrasound, abnormal NIPT results, high risk of serum screening for pregnant women, genetic factors of parents, marriage of close relatives, past adverse pregnancy outcome, and pregnancy at advanced maternal age

Family history abnormalities include parents with mental retardation, ataxia, or epilepsy. Other indications include abnormal fetal karyotypes and fetal karyotypes that do not match FISH test results

^a^Ultrasound abnormalities include structural malformations, abnormal soft markers, fetal growth restriction, and abnormal amniotic fluid volume

^b^Abnormal karyotypes include balanced chromosomal translocations, inversions, duplications, and Aneuploidy

### pCNV fetus results

One of the 17 pCNV cases had a small deletion of the Y chromosome. Sixteen of the cases were chromosome microdeletions or microduplications, including 1 case of Wolf-Hirschhorn syndrome, 1 case of DiGeorge syndrome, 2 cases of “cri du chat” syndrome, 1 case of 7q21 microdeletion, 1 case of 15p11 microdeletion, 1 case of 15q24 microdeletion, 1 case of 15q11 microdeletion, 1 case of 18q22 microdeletion, 1 case of 1q21 microdeletion, 3 cases of 16p11.2 microdeletions, 1 case of 17q12.2 microduplication, and 2 cases of 7q21 microdeletions.

### VOUS fetus results

CMA detected 31 fetuses with VOUS, 15 of which were verified by analyzing the karyotypes of the parents. It was found that the CNV present in 14 cases was inherited from one of the normal parents and that 1 was a new variant. The clinical significance of these CNVs could not be determined. One parent of a fetus with a de novo variant chose to terminate the pregnancy. In cases where VOUS found to be inherited, the classification was still VOUS. As a result, a genetic counselor consulted with the pregnant women and their families to fully explain the uncertainty of the fetal phenotype, and the pregnant woman then decided whether to continue the pregnancy.

According to the prenatal diagnosis indications, the cases were divided into an ultrasound abnormality group and a no ultrasound abnormality group. The ultrasound abnormality group was further divided into a structural deformity group, a soft index abnormality group, and an other ultrasound abnormality group. The results for these various groups were summarized and compared.

### Chromosome and CMA results in the ultrasound abnormality group

#### Ultrasound structural malformation group

Among fetuses with structural malformations, the overall detection rate was 3.81% (8/210) for an abnormal karyotype, 33.33% (3/9) for multiple structural malformations and 2.49% (5/201) for single malformations. The highest chromosome abnormality rate was 33.33% in the multiple malformation group (3/9), followed by 12.5% (1/8) in the hydrocyst of the neck group, 6.67% (1/15) in the varus foot group, and 4.76% (3/63) in the heart malformation group.

The overall detection rates of pCNV were 4.76% (10/210) and 4.97% (10/201) in the single malformation group, and no pathogenic variation was detected in the multiple malformation group. The detection rate of pCNV was the highest in the cleft lip and palate malformation group, at 40% (2/5), followed by 13.33% (2/15) for the neurological malformation group, 5.35% (3/56) for the urinary malformation group, 4.35% (1/22) for the digestive system deformity group, and 3.17% (3/63) for the cardiac malformation group.

The overall detection rates of VOUS were 6.19% (13/210) and 6.47% (13/201) in the single malformation group. VOUS were not detected in the multiple malformation group. In the single malformation group, the highest detection rate of VOUS was 33.33% (5/15) in the nervous system malformation group, followed by 9.09% (2/22) in the digestive system malformation group, 7.94% (5/63) in the cardiac malformation group and 1.78% (1/56) in the urinary system malformation group. See Table 2.

**Table 2:** Results of ultrasound structural malformation group

Prenatal diagnostic indications	n	Abnormal karyotype	Detection rate(%)	PCNVS	Detection rate (%)	VOUS	Detection rate (%)
Single malformation							
Heart malformation	63	3	4.76	2	3.17	5	7.94
Urinary system abnormalities	56	0		3	5.35	1	1.78
Nervous system abnormalities	15	0		2	13.3	5	33.33
Digestive disorders	22	0		1	4.55	2	9.09
Hydrocyst of the neck	8	1	12.5	0			
Foot inversion	4	1	25	0			
Cleft lip and palate	5	0		2	40		
Skeletal abnormalities	11	0		0			
Respiratory system	10						
Pleural effusion	7						
Total	201	5	2.49	10	4.97	13	6.47
Multiple malformations	9	3	33.33	0			
Total	210	8	3.81	10	4.76	13	6.19

#### Ultrasound soft index abnormality group

Among fetuses with abnormal ultrasound soft indicators, the overall abnormal karyotype rate was 1.68% (2/119). The highest chromosomal abnormality rate was in the nuchal translucency (NT) > 3 mm group, at 3.23% (1/31), followed by 2.63% (1/38) in the lateral ventricular dilatation group. Abnormal karyotype were not detected in the other soft index groups.

The overall detection rates of pCNV were 1.68% (2/119) and 5.26% (2/38) in the lateral ventricular dilatation group, and no pCNV was detected in the other abnormal soft index groups.

The overall detection rates of VOUS were 2.52% (3/119) and 3.23% (1/31) in the short nasal bone group and 2.63% (1/38) in the lateral ventricle dilatation group. VOUS were not detected in the rest of the soft index groups. See Table 3.

**Table 3:** Results of ultrasound soft index abnormal group

Prenatal diagnostic indications	n	Abnormal karyotype	Detection rate (%)	PCNVS	Detection rate (%)	VOUS	Detection rate (%)
Lateral ventricle dilatation	38	1	2.63	2	5.26	1	2.63
NT > 3 mm	31	1	3.23	0		1	3.23
Short nasal bone	12					1	8.33
Left ventricular strong echo	13						
Choroid plexus cyst	11						
Echogenic bowel	13						
Eyes wide	1						
Total	119	2	1.68	2	1.68	3	2.52

#### Other ultrasound abnormalities

Among the other ultrasound abnormalities group, the overall rates of an abnormal karyotype were 1.85% (1/54) and 3.33% (1/30) in the fetal growth restriction group. Abnormal karyotypes were not detected in the abnormal amniotic fluid group.

The overall detection rates of pCNV were 3.70% (2/54) and 4.16% (1/24) in the abnormal amniotic fluid group and 3.33% (1/30) in the fetal growth restriction group.

The overall detection rates of VOUS were 7.41% (4/54) and 12.5% (3/24) in the abnormal amniotic fluid group and 3.33% (1/30) in the fetal growth restriction group. See Table 4.

**Table 4:** Results of other ultrasound abnormalities (including abnormal amniotic fluid volume and fetal growth restriction) group

Prenatal diagnostic indications	n	Abnormal karyotype	Detection rate (%)	PCNVS	Detection rate (%)	VOUS	Detection rate (%)
Fetal growth restriction	30	1	3.33	1	3.33	1	3.33
Oligohydramnios	2	0		1	1/2	0	
Polyhydramnios	22					3	13..66
Total	54	1	1.85	2	3.70	4	7.41

#### Comparison of chromosome and CMA results in the different ultrasound abnormality groups

The detection rate of an abnormal karyotype was the highest in the ultrasound structural malformation group, at 3.81% (8/210), followed by the other ultrasound abnormality groups, at 1.85% (1/54). The highest detection rate was in the ultrasound structural malformation group, at 4.76% (10/210). The detection rate of VOUS was the highest in the other ultrasound abnormalities group, at 7.41% (4/54). There were no significant differences in karyotype detection rates among the three groups (*P* > 0.05) and no significant differences in the pCNV and VOUS detection rates (*P* > 0.05; *P* > 0.05). See Table 5.

**Table 5:** Results of ultrasound abnormal group were compared

	Ultrasound soft index abnormal group (*n* = 119)	Ultrasound structural malformation(*n* = 210)	Other ultrasound abnormalities (*n* = 54)	χ^2^	*P* value*
Abnormal karyotype detection rate n (%)	2 (1.68)	8 (3.81)	1 (1.85)	1.468	0.480
PCNVS detection rate n (%)	2 (1.68)	10 (4.76)	2 (3.70)	2.048,	0.359
VOUS detection rate n (%)	3(2.52)	13 (6.19)	4 (7.41)	2.670	0.268

* Pearson Chi-Square significant < 0.05

#### Chromosome and CMA results in the no ultrasound abnormality group

The total chromosome abnormal karyotype rate was 18.75% (12/64) in the no ultrasound abnormality group. The highest chromosomal abnormality rate was in the elderly group, at 66.6% (2/3). The overall detection rate of pCNV was 4.68% (3/64), and the highest detection rate was in the high-risk group, at 33.33% (1/3). The overall detection rate of VOUS was 17.19% (11/64), and the highest detection rate was in the FISH and karyotype discrepancy group, at 50% (1/2). See Table 6.

**Table 6:** Results of no ultrasound abnormal group

Prenatal diagnostic indications	n	Abnormal karyotype	Detection rate (%)	PCNVS	Detection rate (%)	VOUS	Detection rate (%)
NIPT abnormal	25	8	32	1	4	4	16
Advanced maternal age	3	2	66.6				
Abnormal karyotypeof couple	8	1	12.5	0		1	12.5
Fetal Karyotype	11	1	9.09	1	9.09	1	9.09
High-risk screening	3	0	0	1	33.33	1	33
Stillbirth	9					2	22.2
Historyof adverse pregnancy	3					1	33
FISH does not match karyotype	2					1	50
Total	64	12	18.75	3	4.68	11	17.19

### Comparison of detection results between the ultrasound abnormality group and the no ultrasound abnormality group

The detection rate of abnormal karyotypes was 18.75% (12/64) in the no ultrasound abnormality group and 2.87% (11/383) in the ultrasound abnormality group. The detection rate of pCNV was 4.68% (3/64) in the no ultrasound abnormality group and 3.66% (14/383) in the ultrasound abnormality group. The detection rate of VOUS was 17.19% (11/64) in the no ultrasound abnormality group and 5.22% (20/383) in the ultrasound abnormality group. There were extremely significant differences in the detection rate of chromosome karyotype abnormalities. There were also extremely significant differences in the detection rate of chromosome karyotype abnormalities between the two groups (*P* < 0.01), where the nonultrasound abnormal group had a higher detection rate of abnormal karyotypes than the ultrasound abnormal group. There were no significant differences in the detection rate of pCNV (*P* > 0.05). There was an extremely significant difference in the detection rate of VOUS (*P* < 0.01), and the detection rate of VOUS was higher in the nonultrasound abnormal group than in the ultrasound abnormal group. See Table 7.

**Table 7:** Comparison between the no ultrasound abnormality group and the ultrasound abnormality group

	Ultrasound abnormalities group (*n* = 383)	No ultrasound abnormalities group (*n* = 64)	χ^2^	*P* value*
Abnormal karyotype detection rate n (%)	11 (2.87)	12 (18.75)	25.17	< 0.01
PCNVS detection rate n (%)	14 (3.66)	3 (4.68)	0.0022	0.963
VOUS detection rate n (%)	20(5.22)	11 (17.19)	10.38	0.00127

*Pearson Chi-Square significant < 0.05

### Summary of abnormal karyotyping and the PCNVS results in 71 cases

See Table 8.

**Table 8:** Summary of abnormal karyotyping and PCNVS results in 71 cases

Prenatal diagnostic indications	Chromosome results	PCNVS results	Interpretation	VOUS results
Multiplemalformations	47,XN,+ 18	arr[hg19](18 × 3)		
	47,XN,+ 18	arr[hg19](18 × 3)		
	47,XN,+ 18	arr[hg19](18 × 3)		
Abnormal karyotype of couple	46,XN,del(5p15.3)	arr[hg19]5p15.33p15.31 (113,576-9,556,461)× 1	94 Mb	arr(hg19)2p21(43,718,592-44,397,329)×3
Abnormal karyotype	46,XN,del(18)	arr[hg19]18q22.1q23(63.691,770-78,031,728)×1	14.3 Mb	arr(hg19)2p21(43,718,592-44,397,329)× 3
	46,XX,15P +?	arr[hg19]Yq11.23(26,527,669-27,435,790)×0	908.1Kb	
Advanced maternal age	46XY/46,XX?	arr(X)×1–2, (Y)× 0–1		
	47,XN,+ 21	arr(21)×3		
NIPT abnormal	47,XXY	arr(X)×2,(Y)× 1		arr[hg19]22q11.23(23,652,548-25,014,592)× 3
	47,XN,+ 18	arr(18)×3		arr[hg19]1p13.3(109,341,997-109,811,502)× 3
	47,XXY	arr(X)×2,(Y)×1,10q11.23q23.31(52,282,903-91,011,953)× 2 hmz		arr[hg19]10q21.3(68,157,444-68,394,905)×1
	47,XXX	arr(X)×3		arr[hg19]Xp22.33(168,551-675,848))×0 orYp11.32(118,551-625,848)× 0
	47,XN,+ 13	rr(13)× 3		
	47,XN,13- ?	arr[hg19]13q31.1q34(85,516,788-115,107,733)× 1	29.5 Mb	
		arr[hg19]15q11.2q13.1(23,290,787-28,644,578)× 1	5.6 Mb	
	47,XXY	arr(X)×2,(Y)× 1		
	47,XN,+ 18	arr(18)×3		
Heart malformation		arr[hg19]22q11.21(18,648,855-21,800,471)×1	3.1 Mb	arr[hg19]Xp22.31(6,455,151-134,649)×3
		arr[hg19]16q24.3(89,349,122-89,437,985)×1	88.8Kb	arr[hg19]1q21.1(145,406,787-145,760,793)×3
	47,XN,+ 18	arr(18)×3		arr[hg19]4p15.33p15.31(12,959,345-18,763,119)× 3,21q22.12(36,372,117-37,794,593)× 1
	47,XXX	arr(X)×3		arr[hg19]22q11.21(21,029,656-21,800,471)×1
	46,Xn,der(10)t(10:11)(p13;q21)	arr[hg19]10p15.3p14(100,047-10,256,448)×1,11q22.1q25(101,095,537-134,937,416)× 3	Del 10.1 Mb dup 33.8 Mb	arr[hg19]10q21.1(55,848,908-57,027,081)×3
Hydrocyst of the neck	45,X	arr(X)×1		
Fetal growth restriction		arr[hg19]4P16.3P15.32(68, 345–17, 191, 595)×1	17.1 Mb	arr[hg19]5p12p11(45,288,800-46,389,261)× 3
	46,XY[67%]/46,XX[33%]	arr(X)×1–2,(Y)		
Foot inversion	46,XN,der(18)t(2;18)(q36;18q23)	arr[hg19]2q36.1q37.3(221.954,949-242,782,258)×3,18q23(74.065,206-78,013,728)× 1	Dup 20.8 Mb Del l3.9Mb	
NT > 3 mm	47,XN,+ 21	arr(21)× 3		arr[hg19]22q11.23(23,652,548-25,014,592)× 3
Nervous system abnormalities	47,XN,+ 21	arr[hg19]21q11.2q22.3(15,006,457-48,197,372)×2–3	33 Mb	arr[hg19]18p11.32p11.21(136,227-12,092,218)× 2–3
	47,XN,+ 21	arr(21)×3		arr[hg19]15q26.1(90,026,321-90,186,814)× 1
		arr[hg19]7q21.3q21.2(95,425,525-100,874,182)× 1	5.4 Mb	arr[hg19]6q14.1(80,827,070-80,921,191)× 1
		arr[hg19]Yq11.23(26,355,104-27,224,389)×0,16p11.2(28,708,186-29,088,624)× 1	380Kb	arr[hg19]16p13.11(15,058,820–16,538,596)×3
		arr[hg19]16p11.2(28,708,186-29,032,280)× 1	324Kb	arr{hg19}2q13(110,498,141-111,369,264)×1
				arr[hg19]15q11.2(22,770,421–23,286,423)× 1
Urinary system abnormalities		arr[hg19]17q12(34,822,465-36,418,529)×1	1.5 Mb	arr(hg19)18q22.1(65,484,934-66,436,052)× 3
		arr[hg19]17p11.2(16,736,261–20,457,631)×1	3.7 Mb	
		arr[hg19]17q12(34,822,465-36,404,555)×1	1.5 Mb	
Cleft lip and palate		arr[hg19]15q11.2q13.1(23,290,787-28,962,791)× 1	5.3 Mb	
		arr[hg19]5p15.33p15.32(1,708,529-4,597,389)× 1,(X)× 1–2,(Y)×0–1	2.8 Mb	
Oligohydramnios		arr[hg19]22q11.21(18,631,364-21,800,471)×1	3.1 Mb	
High-riskscreening	46,XN,dup(16p11.2)	arr[hg19]Yq11.23(26,527,669-27,472,434)×0,16p11.2q24.3(33,981,749-90,080,142)× 3	56 Mb	arr[hg19]Yq11.23(26,527,669-27,472,434)×0,16p11.2q24.3(33,981,749-90,080,142)× 3
Digestive disorders		arr[hg19]15q24.1q24.2(72,930,195-75,567,135)× 1	2.6 Mb	arr[hg19]2q13q37.3(113,836,347-242,782,258)×3,22q13.2q13.33(43,689,983-51,197,766)× 1
				arr[hg19]Xp11.4(38,117,808-40,223,654)×2,2q13(110,498,141-110,980,295)× 3
Polyhydramnios				arr[hg19]7q31.2(115,293,818-116,777,569)× 3
				arr[hg19]1p36.22(10,327,731-10,686,262)×1
Short nasal bone				arr[hg19]12q24.33(130,475,045-132,484,803)×3
Adverse pregnancy history				arr[hg19]10q21.3(68,141,036-68,551,407)×1
FISH does not match karyotype				arr[hg19]10q23.31q23.32(90,101,722–93,091,030)×3
Pleural effusion				arr[hg19]7p21.3(11,104,500–12,669,251)×3,18q11.2(20,490,086-20,637,568)×1
				arr[hg19]1q43(241,281,153-241,882,720)×3
				arr(hg19)2p21(43,718,592-44,397,329)×3
Total	23	17		31

In each case, karyotype analysis and CMA testing were performed simultaneously. Aneuploidy, abnormal structural abnormalities and chimeras detected by chromosome and CMA were classified as chromosomal abnormalities. Only microdeletions and microduplications detected by CMA were classified as pathogenic copy number variations

Some karyotypes are written with XN (where N = X or Y), as sex of the fetus was not disclosed on prenatal reports

*PCNVs* Pathogenic copy number variations, *VOUS* Variant of uncertain significance

## Discussion

CMA can detect > 1 Kb microdeletions and microduplications with a higher resolution than karyotyping and does not require cell culture. There are also less restrictions on the gestational week (18 to 34 weeks) for analysis. The results of computer analysis used in this approach are objective and reliable, making CMA analysis a valuable approach for chromosomal analysis and prenatal diagnosis [1].

In 2013, the American College of Obstetrics and Gynecology (ACOG) stated that CMA is recommended in place of traditional karyotyping when the fetus has one or more ultrasound abnormalities and requires invasive prenatal diagnosis [2]. Some studies have suggested that CMA can replace traditional karyotype analysis as the first-line detection method for prenatal diagnosis [3]. Our study found that CMA can detect 3.8% more definite genetic abnormalities than karyotype analysis, which is lower than most previous findings [4, 5]. This finding may also be related to the small number of samples in this study.

The detection rate of chromosome karyotype abnormalities in the multiple structural malformation group was higher than that in the single malformation group. Among the types of single malformations, the highest detection rate for karyotype abnormalities was observed in the cervical vesicular tumor group, followed by the foot inversion group and the cardiac malformation group.

Among fetuses with abnormal ultrasound soft indexes, an abnormal karyotype was detected in the NT > 3 mm group and in the lateral ventricle dilation group. No karyotype abnormalities were detected in any of the other soft index groups. Among the other ultrasound abnormal fetuses, an abnormal karyotype was detected in the fetal growth restriction group. Comparisons of the three ultrasound abnormality groups showed no significant differences in the detection rates of karyotype abnormalities.

The chromosomal abnormal karyotype rate was the highest in the ultrasound multiple structural malformations group, with trisomy 18 as the most frequently detected abnormality. Most cases in the multiple structural malformations group had chromosomal abnormalities, but copy number variations were rare.

The chromosomal abnormalities in the single structural malformations group were mainly trisomy 21, sexual chromosomal abnormalities and chromosomal imbalances. The total chromosome abnormal karyotype detection rate was 18.75% in the no ultrasound abnormality group. Compared with the ultrasound abnormality group, the difference in the detection rate of chromosome karyotype abnormalities was extremely significant (*P* < 0.01). Most of the no ultrasound abnormality groups were undergoing prenatal diagnosis due to advanced maternal age, prenatal serological screening, and abnormal noninvasive prenatal testing (NIPT). Chronic chromosomal abnormalities in pregnant women or in one member of a couple increase the incidence of fetal chromosomal abnormalities.

The first round of prenatal screening is mainly aimed at detecting chromosomal abnormalities, and prenatal diagnosis is then performed for those at high risk of chromosomal abnormalities. The target chromosomal abnormalities of NIPT are trisomy 21, 18 and 13. At the same time, the detection rate of sex chromosomal abnormalities is also high. In addition, some microdeletions and microduplications can be detected. In this study, Through prenatal diagnosis, 1 cases were diagnosed as pathogenic microdeletions. The detection rate of PCNVS was 4% (1/25). The detection rate of VOUS was16% (4/25).

This finding indicates that NIPT also has a high detection rate for chromosomal deletions/duplications. Currently, there is a noninvasive NIPTplus detection technology that can detect 17 fetal chromosome aneuploidies and 76 large fetal chromosome deletions/duplications greater than 10 Mb, as well as 100 types of chromosomal microdeletions/duplications. This technology has been launched in some regions. However, NIPT cannot completely replace B-ultrasound and invasive prenatal diagnosis. The abnormal results detected by NIPT still require further invasive prenatal diagnosis for confirmation.

The highest pCNV detection rate was in the structural abnormality group, followed by the other ultrasound abnormality group, and the lowest pCNV detection rate was in the ultrasound soft index abnormality group. No pCNVs were detected in the multiple malformation group. Among the single malformation group categories, the detection rate of pCNVs was highest in the cleft lip and palate group. pCNVs were detected in the lateral ventricular dilatation group in the soft index group and were not detected in the remaining abnormal soft index groups. The highest detection rate of pCNVs was in the ultrasound structural abnormality group, and the lowest pCNV detection rate was in the abnormal soft index group. The detection rate of microdeletions/duplications on chromosome 17 was high in the abnormal urinary system structure group, and for chromosome 16, it was high in the abnormal nervous system group.

Different types of prenatal ultrasound examinations of abnormal fetuses have different detection rates of genetic abnormalities. However, there was no significant difference in the detection rate of pCNVs among the three groups. The detection rate of chromosome karyotype abnormalities was higher in the no ultrasound abnormality group, and the difference was extremely significant. The detection rate of VOUS anomalies was higher in the no ultrasound abnormality group.

CMA testing can detect more genetic microdeletion and microduplication syndromes. However, CMA has a high resolution and also detects many VOUS. This can cause significant stress and even panic among pregnant women and their families, in some cases resulting in the unnecessary induction of labor. Therefore, the indications for CMA testing should be strictly defined, and pregnant women and their families should be fully informed of the possible outcomes and provide consent before CMA is performed.

In 2014, the consensus of Chinese experts was that an abnormal prenatal ultrasound examination in the setting of a normal karyotype analysis is the main clinical indication for using CMA [6]. However, the ACOG and the American Maternal-Fetal Medicine Association’s 2016 guidelines clearly state that CMA can replace chromosome karyotype analysis as a first-line prenatal diagnostic method when ultrasound detects fetal structural abnormalities [7]. Ultrasound structural abnormalities can directly indicate the need for CMA, and the presence of ultrasound soft indicators means either karyotype analysis or CMA can be performed [8–11]. Some studies focused on fetal growth restriction showed that 7.8% of cases had abnormal chromosomal structures or microstructures [12].

Guidelines issued by the Canadian Medical Genetics Association (CCMG) and the Canadian College of Obstetricians and Gynecologists (SOGC) in 2018 recommend rapid CMA screening for isolated structural malformations, multiple malformations, or NT thickening (≥3.5 mm) [13]. Shaffer et al. reported that 68% of fetal abnormalities in multiple systems on ultrasound have a pCNV, which cannot be detected using karyotype analysis [14]. The comprehensive literature shows that when fetal abnormalities are found during prenatal ultrasound examinations, CMA can increase the detection rate of a genetic abnormality by 6 to 7% among fetuses with normal karyotypes [15, 16]. Our study found that CMA could only detect 3.8% more definite genetic abnormalities than karyotype analysis. In our study, not all women with these sonographic findings underwent amniocentesis, which might be relevant from the perspective of selection bias. Maybe our smaller sample size and these selection biases are the reasons for this difference.

In addition, CMA cannot detect the presence or absence of genetic variants and cannot detect single gene diseases. The latest Society for Maternal-Fetal Medicine (SMFM) guidelines recommend that experts conduct a genetic consultation with VOUS patients to review and evaluate all relevant potential genotypes and phenotypes found in existing databases [17]. Some studies have shown that when using fetal nucleic acid samples for whole-genome sequencing or whole-exome sequencing, up to 20 to 30% of fetuses will have genetic abnormalities [18]. Studies such as that of Fu F showed that whole-exome sequencing detected an additional 24% of pathogenic variants, much higher than the 18.2% by karyotype analysis and 8.2% achieved by CMA [19].

Therefore, CMA will still play a key role in the future. With the rapid development of molecular genetics technology and the reduction in detection costs, whole-exome sequencing may become complementary to CMA detection technology in the future to provide the best combination of detection technology in the field of prenatal diagnosis.

## Conclusions

The detection rate of abnormal karyotypes among nonultrasound abnormal cases of prenatal diagnosis was higher than that in the ultrasound abnormality group. For fetuses with abnormal ultrasound structures, CMA can improve the detection rate of fetal genetic abnormalities compared with traditional karyotype analysis. However, nonultrasound abnormalities also have a high detection rate of VOUS abnormalities, so it is necessary to strictly apply the CMA indications.

In this study, the number of cases was small, and the number of cases in some categories is insufficient to allow more precise grouping. Some results found in this study also differ from previous reports. In the future, more cases need to be collected, likely in cooperation with multiple centers, in order to conduct further statistical analysis and to generate a more complete summary of the findings.

## Data Availability

The data that support the findings of this study are available on request from the corresponding author. The data are not publicly available due to privacy or ethical restrictions.

## Abbreviations

CMA: Chromosomal microarray analysis
PCNVs: Pathogenic copy number variations
VOUS: Variant of uncertain significance
SNP: Single nucleotide polymorphism
CNV: Copy number variations
ACMG: American College of Medical Genetics
NT: Nuchal translucency
NIPT: Noninvasive prenatal testing
ACOG: American College of Obstetrics and Gynecology
CCMG: Canadian Medical Genetics Association
SOGC: Canadian College of Obstetricians and Gynecologists
SMFM: Society for Maternal-Fetal Medicine
